# Woman's Sexual Health Knowledge and Needs Assessment in Behavioral Clinics and Shelters in Tehran 

**Published:** 2019-03

**Authors:** Katayoon Tayerih, Zahra Bayat Jozani, Haniyeh Golchehregan, Zohreh Rostam-Afshar, Leila Taj, Sara Ahsani Nasab, Maryam Foroughi, Pegah Mirzapour, Minoo Mohraz, Zohreh Mahmoodi, Zeynab Talebi, Mahbobeh Haji Abdolbaghi

**Affiliations:** 1 Iranian Research Center of HIV/AIDS, Iranian Institute for Reduction of High-Risk Behaviors, Tehran University of Medical Sciences, Tehran, Iran; 2 Social Determinants of Health Research Center, Alborz University of Medical Sciences, Karaj, Iran

**Keywords:** Sexual Health, Shelters, Tehran, Behavioral Clinic, Sexually Transmitted Disease

## Abstract

**Objective:** The aim of this study was to assess the sexual health knowledge among females seeking consultation in behavioral clinics or shelters with emphasis on sexual routs of HIV transmission.

**Materials and methods:** In this study 250 women who have attended behavioral clinics or shelters in Tehran were recruited and a standardized questionnaire which asked about demographics, sexual partner and knowledge about HIV/STDs was used.

**Results:** The median age of our cases was 40.82% and among them 16% were married but lived alone. Among the total 250 cases, 56% (140) were sexually active in the last 30 days, 19.2% (48) had a history of a one-night stand and 2.4% had more than 1 sexual partner. 212 cases answered questions about condom use, 60% (127) of them did not use condoms at all. For knowledge about signs and symptoms related to STDs, 63% believed that abdominal pain has no relation to STDs. Also 44%, 43%, 37%,and 40% believed that dyspareunia, dysuria, malodorous vaginal discharge and change in color of vaginal discharge, respectively had no relation to STDs and 13% of whom presented with these symptoms in the past 30 days had not seek medical evaluation.

**Conclusion:** It is a necessity to emphasize the use of condoms among the male population however in this study it was a challenge to do so because it goes against the government’s campaign of pro-natalism. Improving the knowledge of protected sex should start from the teenage years and at school to have maximum STD prevention planning. Most women in our study did not know about healthy sexual lifestyle and this shows the need of sexual health education before marriage or even at school.

## Introduction

According to the latest statistics of HIV infection, there were approximately 36.7 million people living with HIV of whom 34.5 million were adults and 17 million were female, by the end of 2016. WHO, on the latest global HIV report announced the distribution of new HIV infections in Middle East and North Africa as follows; 41% clients of sex workers and other sexual partners of key populations, 28% people who inject drugs, 18% men who have sex with men and 9% sex workers (1).

Based on the recent report by ministry of health on HIV statistics in Iran, there are 36000 HIV positive registered people of whom 16% are women. In comparison with the same reports in previous years, number of women living with HIV has increased from 3500 to almost 6000 during the last 8 years (2).

Today, the world is committed to end the AIDS epidemic by 2030 (1). The analysis of UNAIDS available data suggests that more than 90% of new HIV infections in central Asia, Europe, North America, the Middle East and North Africa in 2014 were among people from key populations and their sexual partners (3). New researches show that adolescent girls and young women aged 15–24 years old are at particularly high risk of HIV infection, accounting for 20% of new HIV infections among adults globally in 2015, while accounting for 11% of the adult population in general (1).

Although women have greater biological susceptibility to HIV infection (4), it is now well documented in HIV literature that poverty, gender disparity, and social inequality further increase their vulnerability (5). Based on researches and reports, low socio-economic status, working as a sex worker or drug dealer, domestic violence, insufficient access to education and sexual and reproductive health services, are the main factors keeping women at risk of HIV infection.

Regarding stigma toward HIV/AIDS infection and sexual behavior in women, this group pf people do not usually seek for help and is not willing to talk about its needs, and the stigma against HIV/AIDS is a main barrier to early detection and treatment of individuals especially in Middle Eastern and African countries (6, 7). Preventing new HIV infections is a key step toward ending the HIV pandemic as we know (1) and the main key to achieve this objective is having knowledge about HIV transmission and all sexually transmitted diseases (STDs)(8). The increased rate of newly HIV infection in women emphasizes the need of interventions for improving knowledge toward HIV in this population and needs assessment should be the first step. Higher level of sexual and reproductive health knowledge will save women from STDs and unplanned pregnancy.

In addition, although HIV infection rate in women has been trending down around the world, it is not the case in Iran where the number of newly HIV infected women has been increasing. Change in HIV transmission routs from needle sharing to sexual transmission in Iran increases the need for better education of women on the prevention of HIV. Based on the lack of national evidence about women knowledge and behavior on HIV in Iran, we decided to conduct this study to estimate the knowledge about HIV and STDs in women and evaluate the needs assessment of intervention toward SRH knowledge improvement.

## Materials and methods

***Study Location:*** This cross sectional study took place at Tehran, capital city of Iran, in 2015-2017., this study was carried out in three different locations; south, south west and center of Tehran that most drug abuser and females sex workers can be found (8). In addition, the number of women with history of STD who have not been evaluated for HIV or STDs is higher in this parts of the city which made it possible to have access to population at risk of infection with high-risk behaviors (8). These selected locations consisted of two women health services facilities and one HIV primary clinic. The health services facility provides services like, free educational class such as painting, poetry and literatures and life skills classes. Free condom, books and educational brochures are also provided at these facilities.

HIV clinic provides free consult for high-risk behavior people and free HIV and tuberculosis PPD tests. They also provide free condom and syringe for people with high-risk behavior like drug injection or multi sexual partners.

***Sample design and implementation:*** Based on availability and sample size estimates, 250 women were recruited based on the predetermination by the authors, from three different centers. All the participants aged between 18 -65 years old; 100 women from HIV clinic and 150 women from two other sites.

As the survey instrument, a self-administered questionnaire was designed and used in the study of Sexual reproductive health (SRH) needs assessment among mobile and vulnerable population (MVP) communities in Zimbabwe conducted by United Nations Population Fund (UNFPA) and New Consulting Dimension (NEDICO) in 2008 (9). This questionnaire consists of 12 different sections (114 items); baseline information, safe motherhood, mortality profile, family planning, sexual history and function, HIV/AIDS, STIs and violence against women. This questionnaire has been approved and validated for Iranian context by Iranian ministry of health in previous national studies (10).

The participants were informed verbally by the research team members at the study site and by written consent form, based on study main objectives. Patients after signing and approving the consent forms, received the questionnaire. Clinical study was performed as per the guide of Ethics Committee for the Use and Care of Clinical Studies, Tehran University of Medical Sciences, Tehran, Iran (code: IR.TUMS.REC.1394.1616).

***Data Analysis:*** All statistical analyses were performed using SPSS version 16. Continuous variables were reported as mean ± standard deviation (SD), and categorical variables were descripted as percentage. For categorical variables and comparing them, we used Chi-squared test.

## Results

Two hundred and fifty women answered the questionnaire. The Median age of the participants was 40 and Median age of marriage was 19 years old.

Fifty percent of our cases were born and grown up in Tehran. The other majorities belong to Turkish origins of Iran and North Providence 26% and 11%, respectively. The majority of the cases had university degree (69%), bachelor or master, and nine of our cases were illiterate (3.7%). In table 1, we have described marital status of participants. Most of them married or live with spouse (59.2%). Among the participants 231 (92.4%) had history of pregnancy that 30 (13%) of them were unplanned and 17 (7.3%) of them had history of induced abortion.

**Table 1:** Marital status of the participants

**Marital Status and partners **	**Number**	**Percentage**
History of Marriage	206	82.4
No History of Marriage	41	16.4
Married / Live with Spouse	148	59.2
Married / Live with other Sexual Partner	6	2.4
Married / Live without Sexual Partner	40	16
Unmarried / Live with Sexual Partner	7	2.8
Unmarried / Live without Sexual Partner	14	5.6

Among the participants with history of pregnancy 29 persons (12.5%), did not have a history of prenatal or antenatal care and evaluation. Reasons of not having prenatal care are described in table 2. The main reason for not getting prenatal care are financial problems and not having accessible primary care facilities.

**Table 2:** Reasons for not having prenatal care during Pregnancy

**Reason of not having ** **prenatal care**	**Number**		**Percentage**
Financial Problem	16		55.2
I didn’t have access to the Primary care facilities for Prenatal		7	24.2
My Husband didn’t let me go	2		6.9
I afraid of Physician	2		6.9
I didn’t like the behave of Health care providers	1		3.4
Other	1		3.4
Total	29		100

Fatigue (15.2%), abdominal pain (7.5%) and vaginal bleeding (5.1%) are the main complaints of mothers during their pregnancy postnatal complications in women with history of pregnancy and delivering in recent 6 weeks explained in table 3.

**Table 3:** Postnatal complaint in women who have history of pregnancy and childbirth

**Postnatal Complication**	**Number**	**Percentage**
Breast engorgement and pain	37	17.2
Malodor Vaginal Discharge	17	7.9
Severe Vaginal Bleeding	14	6.5
Dysuria	11	5.1
High fever	6	2.8

Among 250 participants, 140 of them had sexual contact during last 30 days. In general, 48 (19.2%) of our cases had history of one nightstand in their sexual contact history.

On the condom use section of our questionnaire, only 212 of 250 case, had answered our questions, that 127 (60%) did not use condom in their sexual intercourse at all.

In table 4, we have explained reasons of not using condom in our study participants.

**Table 4:** Reasons for not using condoms

**Reason of not using condom**	**Number**	**Percentage**
Condom was not easily accessible	12	9.4
My partner doesn’t like it	38	30
It’s difficult to use	23	18.1
I don’t know why	42	33.1
Condom is expensive	7	5.5
Both are HIV +	5	3.9

**Table 5:** Knowledge about HIV infection

**Question**	**Yes** **n (%)**	**I don’t know** **n (%)**
Are you aware of HIV transmission by saliva? (n = 235)	75 (30)	28 (11.2)
Can an HIV+ person work in public places? (n = 234)	156 (66.7)	34 (14)
Can an HIV+ person be a school teacher? (n = 234 )	182 (77.8)	27 (11.5)
Do you know HIV test center locations? (n = 234 )	58 (25)	23 (10)

For sexual contact and violation in sexual intercourse, only 203 individuals answered our questions and 44 of them had unwanted sexual contact during last 30 days. Among this group, 58 (27.2%) persons did not experience orgasm during their sexual contacts and 29 of them did not have any information or knowledge about female orgasm.

Participants' knowledge of HIV is summarized in table 5. For study about the history of STD and the knowledge about its symptoms 229 participants answered the questions (Table 6). Among total 229 participants, 100 of them (43%) have had symptoms of STD during last year and the main complaints were abnormal vaginal discharge (75-32%) and vaginal ulcer (25 -12%). Also about 13% of cases who had STD symptoms did not seek any medical care. The main reasons for this group were similar to prenatal care seeking, financial problems and lack of access to primary health care services. According to the table 6, knowledge about the common symptoms of STDs is less than 50 percent. Only for malodor vaginal discharge, more than 50% of the participants have accepted that this can be symptom of STDs. 

On HIV infection and AIDS questions we evaluated the knowledge of the participants toward HIV/AIDS based on the roots of transmission (Table 7).

We have the less participation on questions for Drug and Alcohol use. For Alcohol use 211 participant answer our questions and 200 (94.8%) said they use alcohol as one or less than one glass drink per week. For history of drug use through inhalation or Intravenous, 173 answered the question and 23 (13.2%) of them said yes. Among people who had history of drug use, 10 women (43%) have been used Cannabis, 15 (65%) Methamphetamines and three of them had history of intravenous drug abuse.

The least participation was in questions related to violence and domestic abuse in women and total 96 individuals had answered this part. Seventy-eight of them (81%) had history of verbal and physical violence, which mainly (73%) was more than one time and the main location was in door. Severe physical injuries fallowed by domestic violence, like bone fracture, has happened in 15 of them (16%).

We had a specific question for violence during sex that 40 (41%) had a positive answer, among them, 71% had this experience with their spouse and others had history of sexual violence with other sexual partners.

## Discussion

The Sexual and Reproductive health (SRH) knowledge should reflect two major topics, Promoting and Protecting. Good SRH knowledge indicates sufficient knowledge of key sexual and reproductive health. The result of our study showed the lack of sufficient knowledge in women about SRH. In addition, women have poor access to the primary health care service.

**Table 6:** Knowledge of women about sexually transmitted disease

**Sign of Sexually transmitted disease**	**Yes** **n(%)**	**No** **n(%)**	**I don’t know** **n(%)**	**Total (n)**
Malodor Vaginal Discharge	122(53.3 )	85(37.1 )	22(9.06 )	229
Turbid Vaginal Discharge	116(50.7 )	93(40.6)	20(8.7 )	229
Dyspareunia	107(46.9 )	101(44.3)	20(8.7 )	228
Dysuria	108(47.2 )	100(43.7)	18(7.9 )	226
Abdominal Pain	63(27.5)	145(63.3)	21(9.2 )	229
Vaginal ulcer or Inflammation	109(47.6 )	99(43.2)	19(8.3)	227
Vaginal itching or burning	106(46.3)	101(40.4)	20(8.7)	227

**Table 7:** Knowledge about HIV transmission roots

**Question**	**Yes** **n(%)**	**I don’t know** **n(%)**
Are you aware of HIV transmission by saliva? (n = 235 )	75 (30)	28 (11.2)
Would you take care of an HIV+ family member? (n = 228 )	112 (49.1)	55 (24.1)
Can an HIV+ person work in public places? (n = 234)	156 (66.7)	34 (14)
Can an HIV+ person be a school teacher? (n = 234 )	182 (77.8)	27 (11.5)
Do you know HIV test center locations? (n = 234 )	58 (25)	23 (10)

Based on the result of our study, different ethnicities, ethical and religious beliefs have important impacts on knowledge and attitude of women in our country.

Given the prevalence of HIV based on global and national statistics and particularly its prevalence among women, needs assessment should be done for this group (11-12). Presence of various ethnic groups living together in the society always leads to some cultural differences (11). As shown in present study, 250 women participated in the survey were of seven ethnicities; among them fifteen women had a hybrid ethnic identity (e.g. a hybrid of Turk and Lor ethnic groups) and two Afghans. When educational programs are developed to improve public health, cultural characteristics, norms, acceptance and also language of relevant ethnic groups should be taken in to account (12). There may be cases in which suitable educational programs are provided but they are not usable and understandable by the audience due to lingual or cultural barriers (13).

On the other hand, because of different educational levels of different populations, too simple or too sophisticated educational programs may lead to boredom or confusion of the audience (14, 15). It is recommended that a simple summary of each educational section being provided in boxes or highlighted or bolded so that the main message of the text be found out only by a brief look at the text (16). 

Sometimes for certain people it is necessary to provide educational materials in their own language to be more understandable, believable and friendlier to them (17).

It is very important to note that marital status of an individual never reveals any information on his/her sexual relationships outside marriage and STD prevention methods should always be accessible and provided to all people in the society regardless of their marital status (18). In addition, in present study 10 percent of subjects were certain about sexual relationship of their spouses with other partner(s). This further highlights the necessity of addressing women educational needs to prevent afflicting with STD via their sexual contact with their spouses. 

Pregnancy is one of the most important time periods in a woman lifetime (19). In this stage, women need more emotional and physical care and it is necessary for all of them to receive required healthcare services. It is a dilemma that in a big city such as Tehran, 12.5% of women participated in the study did not received regular medical care during their pregnancy. Importantly, the most common cause of not attending medical care centers was financial problems and lack of access to those centers. To remove the possibility of HIV transmission from mother to child, it is necessary for all pregnant women to get tested for HIV and be treated as required (20). Taking these actions decreases mother-to-child HIV transmission from 20-40% to less than 1%. In Iran, the first stage of preventing mother-to-child transmission of HIV program has been launched since 2015. Early reports of the program resulted in identification and treatment of some women with HIV diagnosis in the chosen areas (generally low-income suburb ones) (21).

Therefore, to achieve the goal of eliminating mother-to-child HIV transmission, more serious programs should be launched to provide required medical care to women. Indeed, no woman should be excluded from healthcare services during pregnancy for any reason (22).

Present study showed that there are still some cases in which the husband does not allow his wife to attend medical care centers to receive required pregnancy health care services and this emphasizes the need for more extensive communication at family level to decrease pregnancy, labor and delivery risks of complications.

As seen from the survey results, there are still cases of home births usually without any afterbirth care for new mothers which results in high-risk situations. 

In present study, 20% of pregnancies were unintended which emphasizes the importance of regular care for women, appropriate communication with families and participation of men in educational programs. 

Obviously, when pregnancy happens without any planning and preparation, also prenatal care is not received in a regular manner. As seen in table 2, about 50% of pregnant women have attended medical centers with serious complications requiring more examinations.

Also it is seen that about 72% of women have attended medical centers within 6 weeks after childbirth. Attending medical centers during this period is required for appropriate postpartum recovery of new mothers and receiving necessary health information on themselves and their children. Lack of appropriate follow up from medical centers, financial problems and lack of mother's adequate attention to herself and her baby health are among important reasons for inadequate attending medical centers. 

Condom is the most important tool to prevent STDs (23). In present study, it was found that more than 60% of women did not use condom during their sexual activity and the major reason was their partner reluctance to use condom. Unavailability and expensiveness of condom are among the other reasons for not using it. With a brief look at the HIV epidemiological trend in Iran and increased cases of women with HIV and also increased number of people infected with HIV via unprotected sexual contact, it is found that lack of adequate acceptance for using condom in sexual intercourse would have devastating and damaging consequences in terms of afflicting with various STDs including HIV/AIDS. 

On the other hand, current policies in support of population growth contribute to increase limitations of access to condom. Family planning courses in universities are actually cancelled and free condom is no longer provided to the public in relevant health centers (24). If we are not able to develop an appropriate interaction between population growth policies and prevention of STDs and unwanted pregnancies, particularly with respect to financial problems of vulnerable social groups that are basically at the risk of afflicting with these diseases, then risk of these diseases is dangerously increased (25).

Use of condom should become a common health habit (26). Providing education on using condom should begin with the first sexual contacts (27). Besides the health education for young people, education about importance and way of using condom should seriously be taken into consideration (28). Otherwise, a middle-aged man with established sexual habits and probably some degrees of sexual impotency is less likely to use condom in his sexual activity voluntarily (29). 

Forced sexual intercourse also known as marital rape is one of the most important issues in the field of women health (30). Sexual relationship is mutual and both partners have the right to enjoy it to ensure their mental peace, decrease their anxiety and improve their relations (31). In present study, it is found that some women even have no sense of this basic need, i.e. these women not only do not feel any sexual pleasure (27%) but also enjoying sex is meaningless to them.

Obviously, many women lack a healthy sexual relationship as a major factor of reproductive health which needs serious and culturally appropriate education planning for women. STDs are of major importance because of causing multiple problems in sexual relationship, discomfort and sometimes persistent physical complications, reproductive disorders and increased risk of HIV transmission (32, 33). Receiving adequate information on these disorders is highly important so that infected women attend medical centers timely because in many cases relevant signs and symptoms are not severe particularly in women and/or may be confused with their other physiological changes (34).

Thus in reproductive health programs, there should be appropriate education about signs and symptoms of STDs for women (35). As seen in this study, degree of women familiarity with signs and symptoms of STDs was less than 50%. As a result of this, 13% of women with potential symptoms of STDs did not even consult a physician. 

To promote sexual-reproductive health among women, it seems necessary to take advantage of various opportunities to educate them on signs and symptoms of STDs; for example, when they attend gynecology and obstetrics centers, vaccination centers and various urban and rural institutions including libraries, community centers, etc. appropriate education can be provided to them. 

With respect to answers to alcohol and drug consumption questions, 94.8% of those who replied these questions reported their condition as no consumption or at least once a week. However, recent studies in Iran show increased use of alcohol. 

In contrast to alcohol, participants showed no tendency towards answering questions on drug use. Though few number of participants replied these questions, (22 from a total number of 250), few answers showed that various drugs were misused by women. The other major finding is that use of crystal methamphetamine was prevalent among them. Indeed, more than 88% of those who acknowledged using drugs, had a history of crystal methamphetamine consumption. 

One major issue in relation to sexual-reproductive health is domestic violence  (36). Answers to relevant questions showed that all representations of domestic violence including cursing and battering, threatening with cold weapons, intense opposition to personal wills, forced and violent sexual contacts, and such injuries as contusion, bruise, burning and bone fractures due to beating were highly prevalent in families. These violent actions were mostly committed by husbands followed by fathers and brothers.

It is also appeared in some answers that women believed that those violent acts and assaults were natural outcomes of their own deeds and mistakes and accepted them as their punishment. 

Domestic violence is usually the most prevalent form of violence with the most probability of repetition and least probability of report to police. It causes most severe social, mental and economic complications which may result in serious harm to family bases, collapse families and in many cases change people's lives in a destructive way. In addition to women, children are also victims of those violent behaviors (37, 38). 

Such problems as anxiety and sleep disorder at least reported in 50% of the surveyed women suggested their serious psychological disorders. Mother (woman) as the main basis of the family plays a key role in peace and vitality of the home and it is obvious that when women are afflicted with anxiety and depression, it causes a serious damage to family foundation. All health promotion and support programs for women should pay particular attention to specific psychological features of women, promotion of happiness and hope and improvement of individual and collective skills to fight against stressful conditions. Obviously, sexual-reproductive health and psychological health mutually influence each other. Familiarity to symptoms of psychological disorders may help them to attend qualified consultation centers and seek help.

Findings from answers to HIV questions showed that more than 70% of respondents had reasonable knowledge on this disorder and this awareness was especially visible in relation to such issues as appropriate behavior with patients and respecting their confidentiality. But more efforts are needed in relation to the critical issue of preventing mother-to-child transmission (PMTCT). Given the importance of PMTCT and along with the goals of national plan for elimination of HIV transmission to children, need for women education on importance of HIV/AIDS diagnosis and treatment has been highly felt. 

 In the course of the survey, the participants were encouraged and advised to get tested for HIV, but because the stigma associated with AIDS, it was not possible to do HIV test onsite; they were referred to Behavioral Diseases Consultation Center of Imam Khomeini Hospital but most of them said that they would not attend there because of a long journey distance. It should be noted that in Ekbatan neighborhood in District 5 test and consultation were provided onsite and 4 out of 26 subjects, were identified as HIV positive and referred to nearby behavioral diseases centers. Community centers should encourage and recommend doing HIV test and directly provide required facilities and equipment for consultation and quick test of HIV.

## Conclusion

Male participant in reproductive health is so important and It is a necessity to emphasize the use of condoms among the male population however in this study it was a challenge to do so because it goes against the government’s campaign of pronatalism. Improving the knowledge of protected sex should start from the teenage years and at school to have maximum STD prevention planning. Most women in our study did not know about healthy sexual lifestyle and this shows the need of sexual health education before marriage or even at school.
